# Comparison of the Mechanical Properties of Early Leukocyte- and Platelet-Rich Fibrin versus PRGF/Endoret Membranes

**DOI:** 10.1155/2016/1849207

**Published:** 2016-01-06

**Authors:** Hooman Khorshidi, Saeed Raoofi, Rafat Bagheri, Hodasadat Banihashemi

**Affiliations:** ^1^Department of Periodontology, School of Dentistry, Shiraz University of Medical Sciences, Shiraz, Iran; ^2^Department of Dental Materials, School of Dentistry, Shiraz University of Medical Sciences, Shiraz, Iran; ^3^Periodontology Department, Faculty of Dentistry, Shahid Sadoughi University of Medical Sciences, Yazd, Iran

## Abstract

*Objectives*. The mechanical properties of membranes are important factors in the success of treatment and clinical handling. The goal of this study was to compare the mechanical properties of early leukocyte- and platelet-rich fibrin (L-PRF) versus PRGF/Endoret membrane.* Materials and Methods.* In this experimental study, membranes were obtained from 10 healthy male volunteers. After obtaining 20 cc venous blood from each volunteer, 10 cc was used to prepare early L-PRF (group 1) and the rest was used to get a membrane by PRGF-Endoret system (group 2). Tensile loads were applied to specimens using universal testing machine. Tensile strength, stiffness, and toughness of the two groups of membranes were calculated and compared by paired *t*-test.* Results*. The mean tensile strength and toughness were higher in group 1 with a significant difference (*P* < 0.05). The mean stiffness in group 1 was also higher but not statistically significant (*P* > 0.05).* Conclusions.* The results showed that early L-PRF membranes had stronger mechanical properties than membranes produced by PRGF-Endoret system. Early L-PRF membranes might have easier clinical handling and could be a more proper scaffold in periodontal regenerative procedures. The real results of the current L-PRF should be in fact much higher than what is reported here.

## 1. Introduction

Periodontal reconstruction is the ideal goal of periodontal treatment and since 1970, many researches led to developing various methods to achieve it. Among these methods, guided tissue regeneration (GTR) and guided bone regeneration (GBR) use barrier membranes to separate the periodontal ligament and bone from the epithelium and connective tissue which allow the former to regenerate the defects [1]. Recently, various growth factors have been studied in periodontal regeneration [2] and it is indicated that they might strongly alter the healing process [3]. A new method in this field is using concentrated platelet products which are the source of autologous platelet derived growth factors and transforming growth factors [4].

Among various concentrated platelet products, preparation rich in growth factor (PRGF) is an autologous platelet-rich plasma product which accelerates local release of growth factors and bioactive proteins following its activation. With various formulations, this product can be used in form of liquid or in form of clot as a membrane which is a biocompatible, dense, and elastic membrane [5]. The new form of concentrated platelet is platelet-rich fibrin (PRF) that can be used directly as a clot or as a strong membrane after compression [6, 7]. PRF as a membrane has shown slow release of growth factors such as vascular endothelial growth factor (VEGF), transforming growth factor (TGF-*β*), and platelet derived growth factor (PDGF) for at least 7 days in vitro [8]. Leukocyte- and platelet-rich fibrin (L-PRF) can be considered as a second-generation platelet concentrate. It forms a strong fibrin matrix with a complex three-dimensional architecture, in which most of the platelets and leucocytes from the harvested blood are concentrated [9]. Platelet-rich fibrin membranes can be used in various regenerative treatments [10, 11] to accelerate healing, to progress the regeneration process, and also as a scaffold in tissue engineering.

Besides scientific evidences about efficacy, for selection of an appropriate membrane, there are other important parameters including mechanical properties and clinical handling [12, 13]. Mechanical characteristics of the membrane may affect the final results of GBR [14]. Tensile strength of a material when sutured may affect the clinical result of following healing [15]. Moreover strong mechanical characteristics of a scaffold provide a more suitable support for regeneration [16].

It is reported that increasing fibrinogen concentrates and adding calcium chloride increase the adhesion and tensile strength of fibrin clot [17]. It is also indicated that increasing thrombin and fibrinogen may increase the stiffness of fibrin matrix [18].

To the best of authors' knowledge, a comparison of mechanical characteristics of PRF and PRGF membranes is missing in previous studies. The goal of this study was to compare the mechanical properties of early L-PRF and the PRGF membranes. The null hypothesis was that there is no difference between mechanical properties of early L-PRF and PRGF membranes.

## 2. Materials and Methods

In this experimental study, 20 cc venous blood was obtained from 10 healthy volunteer males with age range of 25 to 35 years. The exclusion criteria were suffering from a known systemic disease, history of taking any anticoagulant medication, smoking, and history of taking any medicine in the past 3 months.

### 2.1. Preparing a Mold

**Figure 1:**
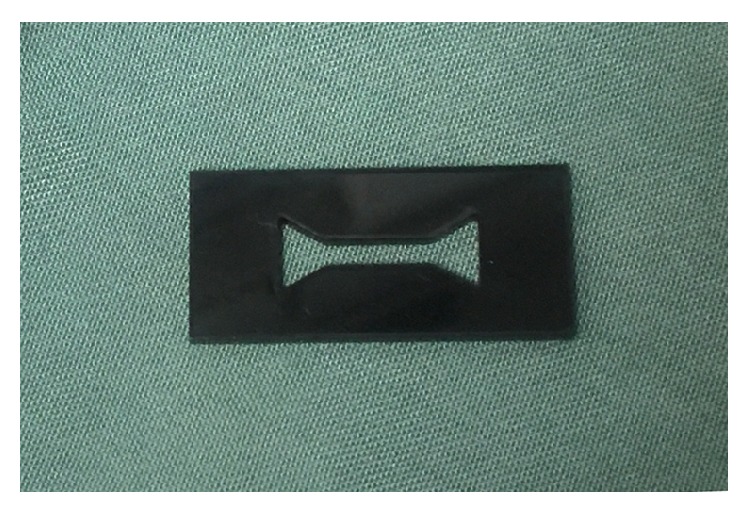
The dog-bone-shape plexiglass mold.

A specially designed plexiglass mold was fabricated to make the fibrin specimens identical in size, volume, and figure, following a modification of the dog-bone-shape mold in Alston's study [17]. The thickness of the mold was 2 mm and the width was 2 mm in the narrow middle part and 6 mm in the larger ends. The total volume of the mold was 104 mm^3^. The narrow neck provided the weakest point where the specimen would break (Figure 1).

### 2.2. Blood Collection

After obtaining informed consent approved by the ethical committee of Shiraz University of Medical Science (Grant number 92-01-03-6162) from all donors, 20 cc venous blood was collected by sterile syringe. 10 cc was placed in a dry sterile tube specific for PC-02 machine and the rest was divided into two 5 cc blood samples placed in two tubes containing 0.5 cc 3.8% concentrate of sodium citrate as anticoagulant specific for PRGF-Endoret system.

### 2.3. Preparing the Membranes

**Figure 2:**
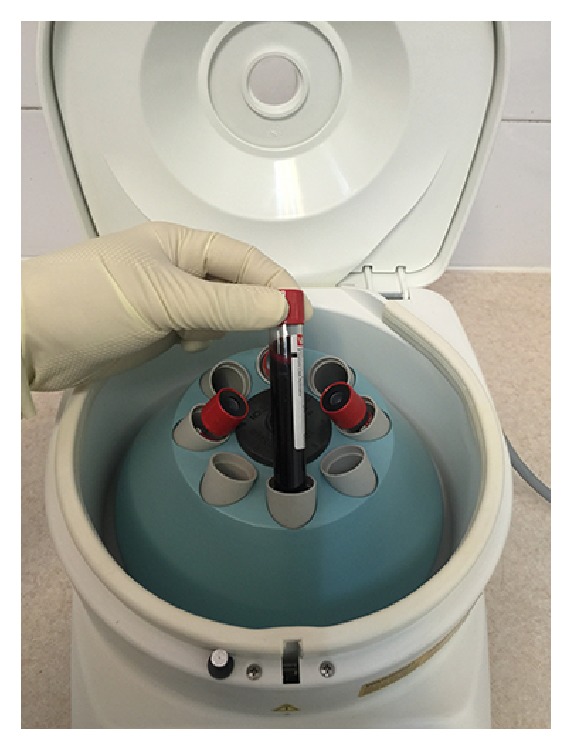
Tube containing blood in the centrifuge machine.

**Figure 3:**
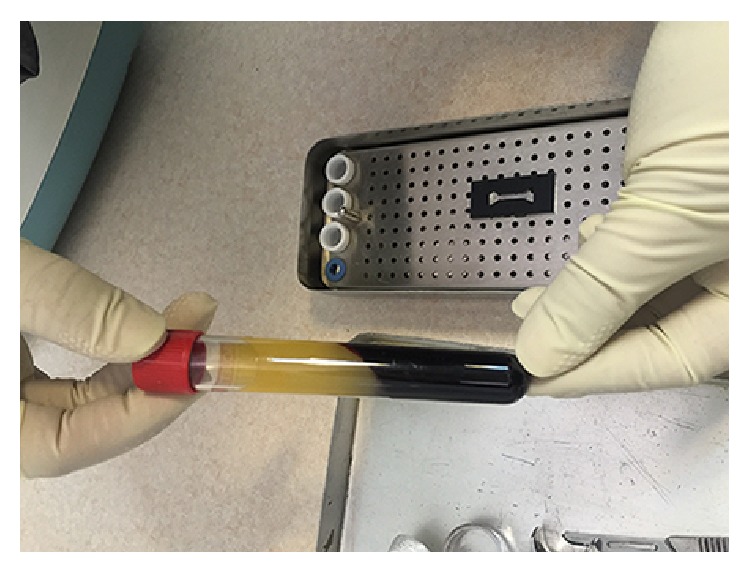
Tube containing early L-PRF after centrifuge.

**Figure 4:**
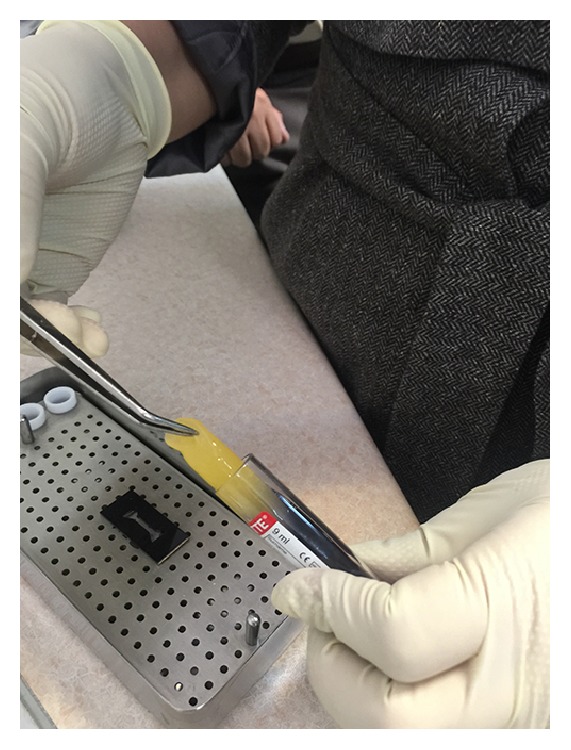
Removing the early L-PRF from the tube.

**Figure 5:**
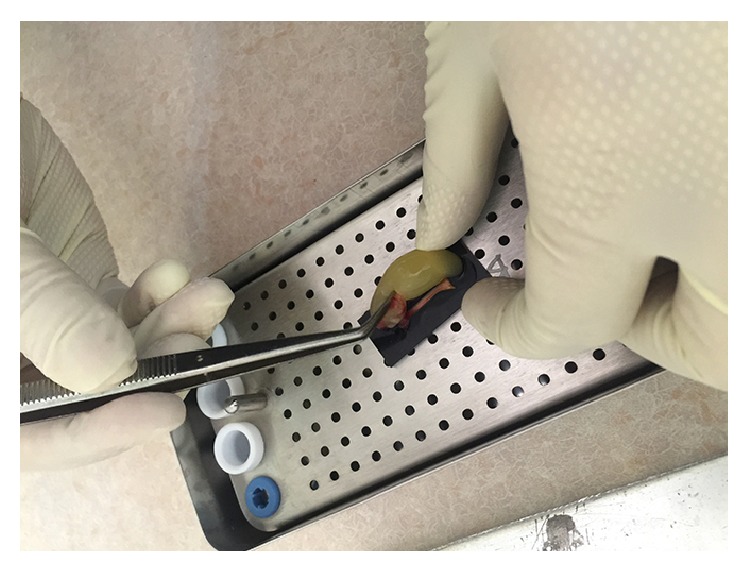
Placing the early L-PRF clot into the mold.

**Figure 6:**
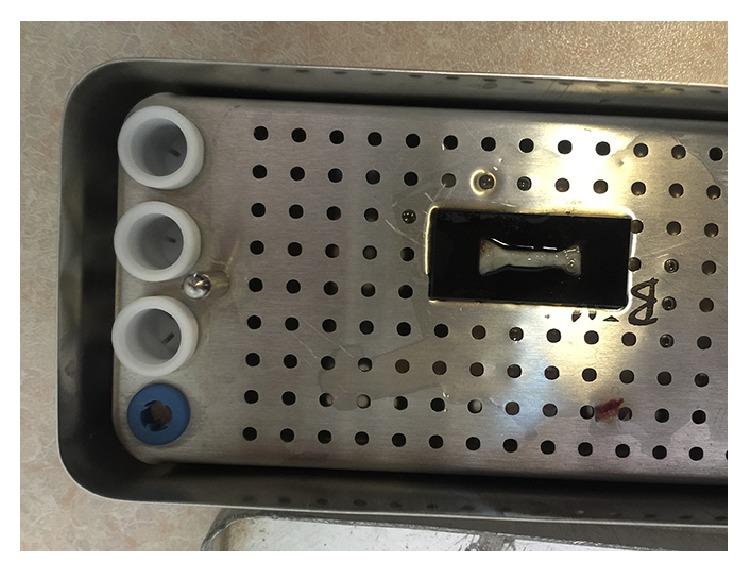
A fibrin specimen in the mold.

**Figure 7:**
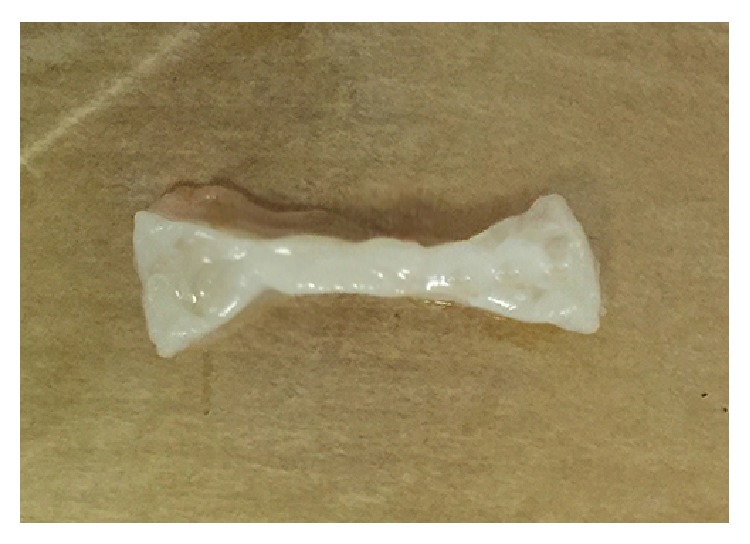
A formed specimen.

**Figure 8:**
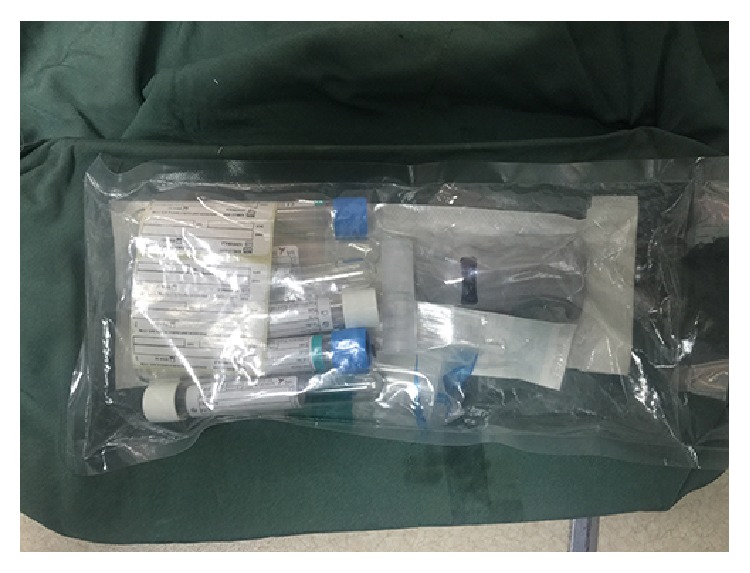
A BTI kit.

**Figure 9:**
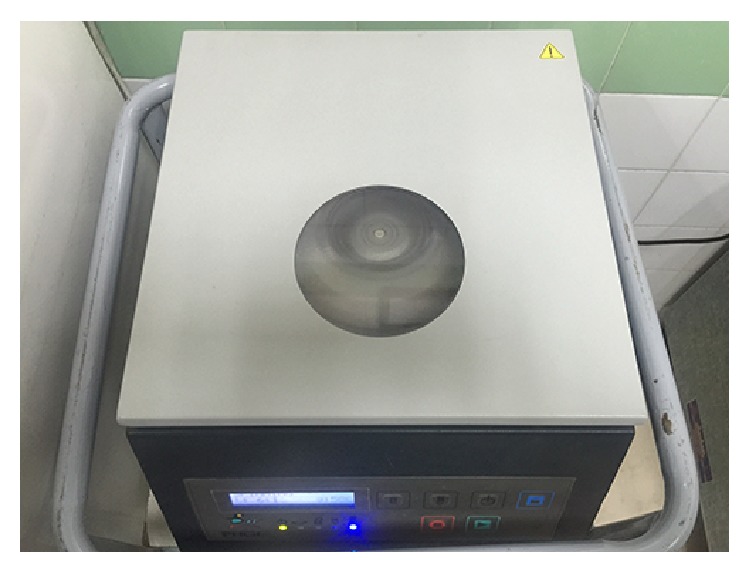
BTI centrifuging machine.

**Figure 10:**
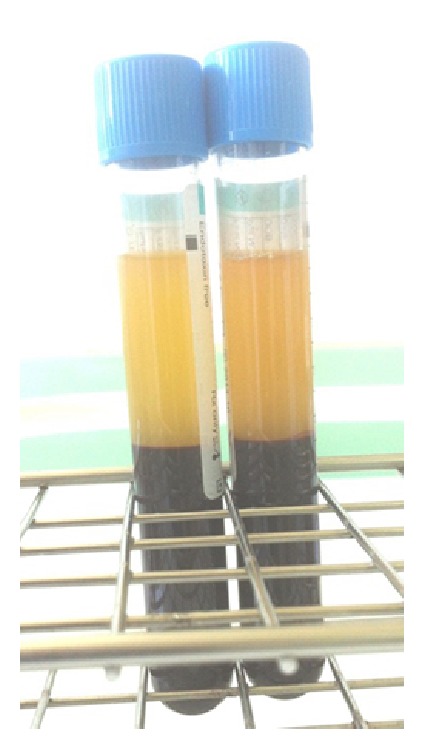
Tubes containing PRGF after centrifuge.

**Figure 11:**
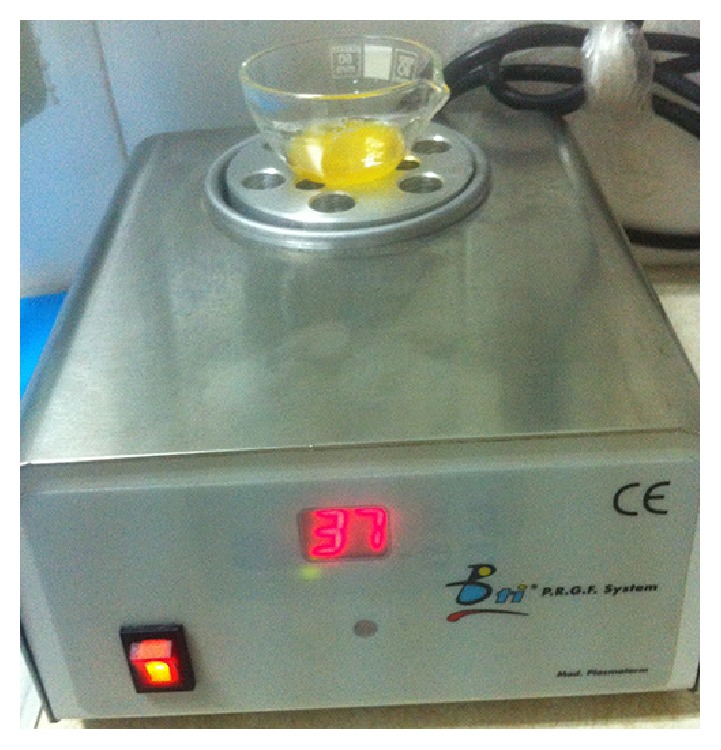
Mixture of the platelet-rich plasma and the activator on the incubator.

**Figure 12:**
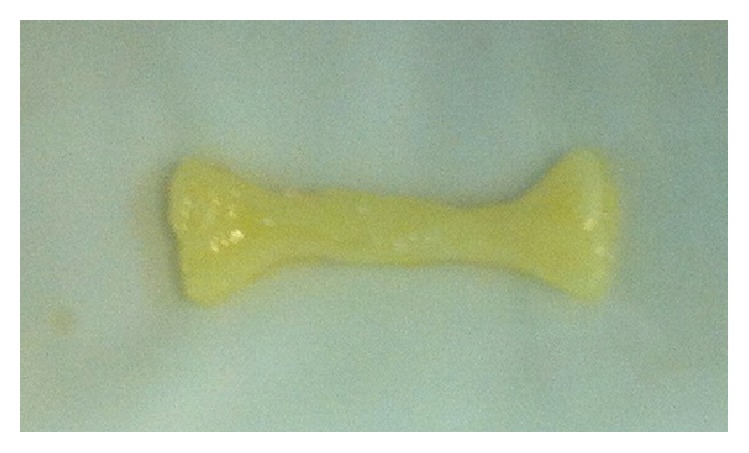
A formed specimen.

Platelet-rich membranes were obtained by two different protocols:

 The first one was producing early L-PRF [7] by PC-02 machine (Process Ltd., Nice, France) in which the tube that contained blood was centrifuged immediately after blood collection in speed of 400 gr for 10 min [19] (Figure 2). The outcome was a fibrin clot containing platelets in the middle of the tube, between acellular plasma at the top and the red blood cell layer at the bottom (Figure 3). This clot was removed from the tube (Figure 4) and the attached red blood cells were scraped off and discarded. The early L-PRF clot was then placed in the mold (Figure 5) which was placed on the grid in the PRF Box [20] (Process Ltd., Nice, France) (Figure 6) and covered with the compressor and lid. After 10 min the formed early L-PRF membrane was prepared (Figure 7).
 The second protocol was performed using PRGF-Endoret system (BTI, Spain) (Figure 8). The two 5 cc tubes were centrifuged in speed of 400 gr for 8 min with BTI centrifuge machine (BTI, Spain) (Figure 9). Then each tube contained red blood cell at the bottom and plasma at the top with a thin layer of WBC in the middle (Figure 10). The inferior half of the plasma which was rich in platelets and growth factors was removed by plasma transfer device 2 (PTD2) (BTI, Spain) and placed in another tube. As the manufacturer instructions, 0.05 mL PRGF-Endoret activator per 1 mL plasma was added and then placed on incubator at 37°C for 30 min to obtain the clot (Figure 11). The clot was placed in the mold and after 10 min the formed membrane was obtained (Figure 12).

### 2.4. Tensile Test

**Figure 13:**
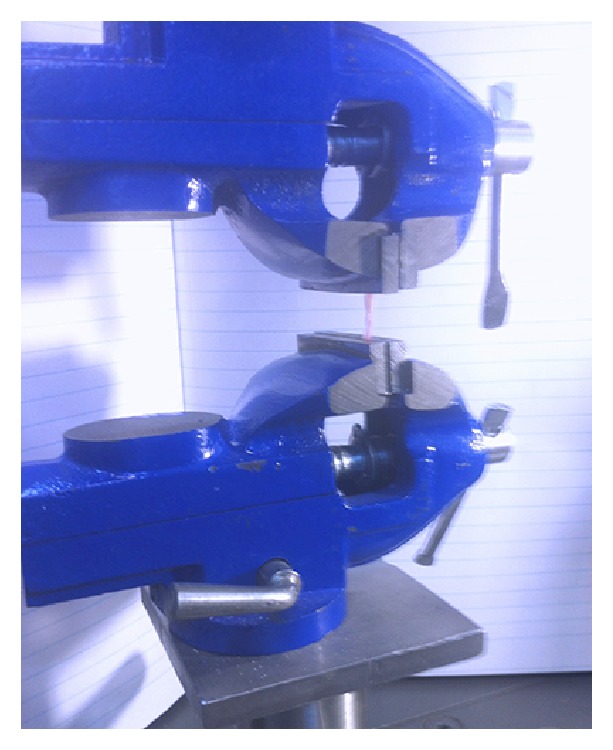
The universal testing machine.

Tensile test was performed using universal testing machine (Zwick/Roll Z020, Zwick GmbH & Co., Germany) (Figure 13). The larger ends of the dog-bone shape specimen were held with the clips of the machine without any tension. Tensile loading was applied at a cross head speed of 2 mm/min; the maximum load at specimen failure was recorded and tensile strength was calculated using following formula: *S* = *F*/*A*, where *F* is maximum force (N) and *A* is unit area (m^2^).

Stress-strain curve was recorded with test Xpert II software simultaneously. Stiffness of the specimen (modulus of elasticity) was obtained by stress/strain and the total area under the curve designated as toughness of the specimens.

### 2.5. Data Analysis

Data were collected and analyzed using SPSS version 16; Student *t*-test was used to compare the groups: the early L-PRF as group 1 and the PRGF-Endoret system as group 2.

## 3. Results

The results of all tests for two groups are summarized in Table 1.

Tensile strength of early L-PRF group with mean value of 0.20 ± 0.06 MPa was significantly higher than PRGF group with mean value of 0.14 ± 0.07 MPa (*P* = 0.049). Early L-PRF group was slightly stiffer than PRGF group but was not statistically significant (*P* = 0.69). Toughness of early L-PRF group was significantly higher than PRGF group (*P* = 0.001).

## 4. Discussion

This study experienced that the mechanical properties of early L-PRF membranes are stronger than the PRGF-Endoret membranes.

Platelet-rich fibrin membrane releases various growth factors such as PDGF, TGF-*β*, and VEGF slowly [21] and its supportive fibrin matrix plays an important role in its therapeutic effects [6]. The potential of platelet-rich membrane in accelerating regeneration has led to its application in various regenerative treatments like sinus floor elevation, ridge augmentation, socket preservation, root coverage, intrabony defects, and furcation defects [22–31]. It has been shown that fibrin membranes could be better scaffolds for proliferation of periosteal and osseous cells than collagen membranes in vitro [32, 33]. The membranes that are used in regenerative procedures should have strong mechanical properties to protect blood clot and healing process [13]. As a scaffold, they provide better support against forces from infiltrating cells and adjacent tissues [16, 34].

The specimens of this study were selected from healthy male individuals with the age range of 25–35 to prevent possible bias from varieties in blood components of different sexes, ages, and systemic conditions. These issues were not considered together in the previous studies on fibrin clots [17, 35, 36]. The dog-bone-shape mold was used to make the specimens identical in size, volume, and shape. It was a modification of Alston's method [17], since the volume of clot we could obtain and consequently the volume of our specimen were lesser than Alston's study. Mechanical measurements were performed by universal testing machine as some other studies [17, 35–38].

According to the results of our study, the tensile strength, stiffness, and toughness of early L-PRF membranes were higher than the PRGF-Endoret membranes though the stiffness difference was not significant. This result may be due to their structural differences which may be affected by some factors like their differences in polymerization. The mode of polymerization has significant effects on mechanical properties of fibrin matrix [39]. This is consistent with the studies that evaluated their polymerization and internal structure [9, 39]. The last stage of clotting, in which fibrinogen is converted to fibrin, can be accelerated by adding calcium chloride [40]. In PRGF-Endoret system, calcium chloride is used to initiate the last coagulation stage; then sudden fibrin polymerization occurs [39]. Therefore the fibrin matrix is immature and most of the fibrils are thin [9]. On the other hand, a slow and natural polymerization occurs during the centrifuge process in L-PRF producing method. The fibrin fibrillae can be assembled in 2 different biochemical architectures during gelling process: condensed tetramolecular or bilateral junctions and connected trimolecular or equilateral junctions [39]. PRGF mostly have the bilateral junctions which are weaker than the equilateral junctions [9] that are mostly found in L-PRF. This provides a flexible and elastic fibrin network [39]. L-PRF has thick fibers and strong matrix [9].

The density of the final fibrin matrix is another important factor that has an influence on the mechanical properties [41] and fibrinogen concentration affects this parameter. Fibrinogen mostly originates from the ∝-granules of the platelet in PRGF so the final fibrin has low density, while the circulating fibrinogen present in L-PRF strengthens the final fibrin matrix [9]. Alston et al. (2007) and Duong et al. (2009) indicated that increase in fibrinogen concentration makes the final fibrin matrix stronger [17, 18].

Another difference of these two membranes is the presence of large quantities of leukocytes in L-PRF and lack of them in PRGF. Some studies indicated that leukocytes have a key role in immune regulation, anti-infection properties [42–45], and angiogenesis [46] in platelet-rich concentrates. On the other hand, some authors claim that leukocytes may destroy the extracellular matrix of fibrin by the anti-inflammatory effects of proteases and hydrolase; therefore they suggest removing the leukocytes from platelet-rich concentrates to prevent their negative effects on autologous fibrin formation [5, 47]. The interaction of platelets and leukocytes in platelet-rich concentrates is not completely analyzed; they may also show synergistic effect [9]. Therefore the negative effects of leukocytes on fibrin matrix are controversial yet and our results suggest that these effects are not significant.

In producing the early L-PRF, no anticoagulant is employed but, in PRGF, sodium citrate is used. This difference of these two methods may affect the fibrin matrix. Kingston showed that high concentration of sodium citrate in blood samples decreases the ionized calcium of plasma leading to decrease in platelet accumulation and fibrinogen binding [48]. However, it is not obvious that 0.5 cc 3.8% concentration of sodium citrate can have such an effect on PRGF matrix and controlled studies are needed to confirm this issue.

The room temperature during the process and the speed of centrifuge were the same in both groups but the duration was less in PRGF. Perez et al. showed that longer duration of centrifuge increases the platelet recovery [49] so this may affect the plasma components and final fibrin matrix properties.

Parameters such as manufacturing property of the blood collecting tubes and the pressure applied during the process do not affect the biomaterial structure [8].

It should be noted that we used the early protocol (3000 rpm, 10 minutes) to produce L-PRF, while since years the 2700 rpm/12 minutes protocol is mostly used that gives much better polymerized L-PRF and therefore stronger membranes than the 3000 rpm/10 min protocol. The real results of the current L-PRF should be in fact much higher than what is reported here. However the material we used is adequate for the production of a good quality original L-PRF (early protocol). The original L-PRF system now exists only in one CE/FDA cleared form that is termed Intra-Spin L-PRF (Intra-lock, Boca Raton, FL, USA), and, legally, it is the only kit/system allowed in Western countries (CE/FDA).

## 5. Conclusion

Considering the limitations, this study showed that early L-PRF membranes have stronger mechanical properties than platelet-rich membranes obtained by PRGF-Endoret system. Probably, they have easier clinical application and handling, and they may also be stronger during suturing and provide more supportive scaffold in periodontal regeneration. The real results of the current L-PRF should be in fact much higher than what is reported here.

## Figures and Tables

**Table 1:** Mean values and standard deviation (±SD) for all tested properties in the two groups.

Measured values	Group	Mean ± SD	*P* value
Tensile strength (MPa)	1	0.20 ± 0.06	0.049
	2	0.14 ± 0.07	

Modulus of elasticity (MPa)	1	0.13 ± 0.07	0.69
	2	0.11 ± 0.09	

Toughness (Joule/m^3^)	1	1.87 ± 0.61	0.001
	2	0.81 ± 0.53	
